# Rapid Manufacturing of Highly Cytotoxic Clinical-Grade SARS-CoV-2-specific T Cell Products Covering SARS-CoV-2 and Its Variants for Adoptive T Cell Therapy

**DOI:** 10.3389/fbioe.2022.867042

**Published:** 2022-04-04

**Authors:** Agnes Bonifacius, Sabine Tischer-Zimmermann, Maria Michela Santamorena, Philip Mausberg, Josephine Schenk, Stephanie Koch, Johanna Barnstorf-Brandes, Nina Gödecke, Jörg Martens, Lilia Goudeva, Murielle Verboom, Jana Wittig, Britta Maecker-Kolhoff, Herrad Baurmann, Caren Clark, Olaf Brauns, Martina Simon, Peter Lang, Oliver A. Cornely, Michael Hallek, Rainer Blasczyk, Dominic Seiferling, Philipp Köhler, Britta Eiz-Vesper

**Affiliations:** ^1^ Hannover Medical School, Institute of Transfusion Medicine and Transplant Engineering, Hannover, Germany; ^2^ Deutsche Gesellschaft für Gewebetransplantation, Hannover, Germany; ^3^ Department I of Internal Medicine, Faculty of Medicine and University Hospital Cologne, Center for Integrated Oncology Aachen Bonn Cologne Duesseldorf, University of Cologne, Cologne, Germany; ^4^ Faculty of Medicine and University Hospital Cologne, Translational Research, Cologne Excellence Cluster on Cellular Stress Responses in Aging-Associated Diseases (CECAD), University of Cologne, Cologne, Germany; ^5^ Department of Pediatric Hematology and Oncology, Hannover Medical School, Hannover, Germany; ^6^ Miltenyi Biotec B.V. & Co. KG, Bergisch Gladbach, Germany; ^7^ Department of Pediatric Hematology and Oncology, University Children’s Hospital, University of Tuebingen, Tuebingen, Germany; ^8^ Faculty of Medicine and University Hospital Cologne, Clinical Trials Centre Cologne (ZKS Köln), University of Cologne, Cologne, Germany; ^9^ German Centre for Infection Research (DZIF), Partner Site Bonn-Cologne, Cologne, Germany; ^10^ Miltenyi Biomedicine GmbH, Bergisch Gladbach, Germany

**Keywords:** adoptive T cell therapy, antiviral T cells, immunotherapy, SARS-CoV-2, COVID-19

## Abstract

**Objectives:** Evaluation of the feasibility of SARS-CoV-2-specific T cell manufacturing for adoptive T cell transfer in COVID-19 patients at risk to develop severe disease.

**Methods:** Antiviral SARS-CoV-2-specific T cells were detected in blood of convalescent COVID-19 patients following stimulation with PepTivator SARS-CoV-2 Select using Interferon-gamma Enzyme-Linked Immunospot (IFN-γ ELISpot), SARS-CoV-2 T Cell Analysis Kit (Whole Blood) and Cytokine Secretion Assay (CSA) and were characterized with respect to memory phenotype, activation state and cytotoxic potential by multicolor flow cytometry, quantitative real-time PCR and multiplex analyses. Clinical-grade SARS-CoV-2-specific T cell products were generated by stimulation with MACS GMP PepTivator SARS-CoV-2 Select using CliniMACS Prodigy and CliniMACS Cytokine Capture System (IFN-gamma) (CCS). Functionality of enriched T cells was investigated in cytotoxicity assays and by multiplex analysis of secreted cytotoxic molecules upon target recognition.

**Results:** Donor screening via IFN-γ ELISpot allows for pre-selection of potential donors for generation of SARS-CoV-2-specific T cells. Antiviral T cells reactive against PepTivator SARS-CoV-2 Select could be magnetically enriched from peripheral blood of convalescent COVID-19 patients by small-scale CSA resembling the clinical-grade CCS manufacturing process and showed an activated and cytotoxic T cell phenotype. Four clinical-grade SARS-CoV-2-specific T cell products were successfully generated with sufficient cell numbers and purities comparable to those observed in donor pretesting via CSA. The T cells in the generated products were shown to be capable to replicate, specifically recognize and kill target cells *in vitro* and secrete cytotoxic molecules upon target recognition. Cell viability, total CD3^+^ cell number, proliferative capacity and cytotoxic potential remained stable throughout storage of up to 72 h after end of leukapheresis.

**Conclusion:** Clinical-grade SARS-CoV-2-specific T cells are functional, have proliferative capacity and target-specific cytotoxic potential. Their function and phenotype remain stable for several days after enrichment. The adoptive transfer of partially matched, viable human SARS-CoV-2-specific T lymphocytes collected from convalescent individuals may provide the opportunity to support the immune system of COVID-19 patients at risk for severe disease.

## Introduction

Despite the approval of several vaccines against SARS-CoV-2, there is a high medical need for further therapeutic options to facilitate convalescence from the infection and prevent a severe course of the disease. Early treatment strategies for COVID-19 patients include immunomodulatory approaches such as dexamethasone, antiviral substances such as remdesivir, antimalarial drugs, and monoclonal antibodies or convalescent plasma (COVID-19 Treatment Guidelines Panel, 2021). The appearance of new variants (variant of concern, VOC) calls for new therapeutic strategies as increased clinical severity of COVID-19 and even vaccine evasion may pose additional problems (World Health Organisation, 2021). Especially the efficacy of treatment with convalescent plasma is in question since the target of neutralizing antibodies, the receptor-binding domain (RBD) is increasingly mutated in VOCs and immune escape variants may emerge (Cameroni et al., 2021; Focosi et al., 2021; Wang et al., 2022). Recently, the antiviral drugs Molnupiravir (Lagevrio) and Nirmatrelvir (Paxlovid), inhibiting viral replication, have been authorized for treatment of COVID-19 by the FDA^1^ ^2^, while the European Medicines Agency is currently evaluating their marketing authorization application^3^. However, these drugs have to be administered at very early time point of infection and come with potentially severe side effects, therefore their application has to be carefully evaluated and is limited to patients at risk for severe disease. Moreover, data regarding the antiviral effect of Nirmatrelvir on Omicron variant (B.1.1.529) are not yet available. During SARS-CoV-2 infection and subsequent recovery, SARS-CoV-2-specific T cells emerge as part of the adaptive immune response mediating control of infected cells, viral clearance, and possibly long lasting protective immunity (Chen and John Wherry, 2020; Bertoletti et al., 2021; Pilz et al., 2022).

Throughout recovery and convalescence, significant numbers of SARS-CoV-2-specific memory T cells reactive against Spike (S), Nucleocapsid (N), and Membrane (M) proteins of SARS-CoV-2 are detectable (Peng et al., 2020; Bonifacius et al., 2021). Particularly in convalescent individuals recovering from mild or even asymptomatic disease, a robust, broad and highly functional T cell response can be detected suggesting effective protection from SARS-CoV-2 reinfection (Bonifacius et al., 2021; Gussarow et al., 2021; Le Bert et al., 2021; Marcotte et al., 2022). In contrast to neutralizing antibodies, antiviral T cells developed during SARS-CoV-2 infection with wild type or earlier VOCs, were shown to efficiently recognize antigens from Omicron variant (Gao et al., 2022). On the other hand, some patients do not mount an appropriate, efficient T cell reaction but exhibit inadequate immune responses (Jarjour et al., 2021). Patients with severe COVID-19 generally develop lymphopenia, disruption of the T cell compartment, and CD8^+^ memory T cell exhaustion (Bacher et al., 2020; He et al., 2021; Vigón et al., 2021). Reduced T cell counts and an increased granulocyte to lymphocyte ratio correlate with disease severity and are early predictive markers of an adverse course (Kong et al., 2020; Liu et al., 2020). In patients with severe and advanced disease, misled innate and adaptive immune cells can cause a hyperinflammatory state, often resulting in endothelial inflammation, hypercoagulation, and end-organ damage (García, 2020). The adoptive transfer of SARS-CoV-2-specific T cells isolated from convalescent individuals might therefore support the patient’s immune system allowing protective T cell immunity to be restored and subsequent viral clearance in these patients (Hanley et al., 2020).

The concept of adoptive T cell therapy has been developed for viral infections in immunosuppressed patients after allogeneic stem cell (HSCT) and solid organ transplantation (SOT). Refractory viral infections due to Cytomegalovirus (CMV), Adenovirus (AdV), and Epstein-Barr virus (EBV) after HSCT represent life-threatening conditions due to the insufficient endogenous T cell response of the recipient and lack of effective treatment options. However, the concept of adoptive T cell therapy not only applies to patients with transplantation history but also to those generally immunocompromised due to a variety of underlying diseases. In detail, progressive multifocal leukoencephalopathy (PML), caused by reactivation of JC polyomavirus (JCV), occurs in immunocompromised patients, and is associated with high morbidity and mortality mainly because there are no targeted therapies available (Brew et al., 2010; Cortese et al., 2021). Hence, the reconstitution of T cell immunity by adoptive transfer of virus-specific T cells is a promising approach (Muftuoglu et al., 2018; Steinhardt et al., 2020; Berzero et al., 2021; Hopfner et al., 2021).

The repertoire of SARS-CoV-2-specific T cells in COVID-19 patients convalescent from mild disease covers a broad range of viral antigens including antigens from different VOCs. In contrast, in the majority of hospitalized patients reduced functionally active antiviral T cells are observed (Bergamaschi et al., 2021; Bonifacius et al., 2021; He et al., 2021). Adoptive transfer of virus-specific T cells in different settings appears safe and effective (Withers et al., 2017). It therefore can be hypothesized that the transfusion of viable donor-derived SARS-CoV-2-specific T cells collected from convalescent individuals will support the immune systems of individuals at risk during an early course of the disease (Hanley et al., 2020). Due to the possibility of rapid deterioration of the patient, quick turnaround times in production, and logistics of T cell substances are mandatory. The CliniMACS Cytokine Capture System (CCS) IFN-gamma allows for rapid generation of antiviral T cell products in sufficient number and purity within 16 h after leukapheresis using CliniMACS Prodigy instrument. Moreover, since the SARS-CoV-2-specific peptide pool PepTivator SARS-CoV-2 Select contains peptides derived from structural as well as non-structural proteins and since the majority of these epitopes are identical between wild type SARS-CoV-2 and circulating VOCs, antiviral T cell products generated using PepTivator SARS-CoV-2 Select display a broad antigenic specificity covering epitopes from both, wild type SARS-CoV-2 and VOCs. By screening the mutations of the currently circulating VOCs we found in Alpha 1-, in Delta 2- and in the Omicron subtype 5 out of 88 peptides of the PepTivator SARS-CoV-2 Select being affected by amino acid mutations of the respective Virus subtype^4^. Thus, even the currently circulating Omicron variant with the highest number of mutations in different proteins is matched by > 90% of peptides contained in PepTivator SARS-CoV-2 Select. Three out of five affected peptides of the PepTivator SARS-CoV-2 Select are solely altered in their periphery when compared to the Omicron Virus subtype sequence, suggesting that these peptides will still be presented on the respective HLA alleles. Moreover, the HLA subtypes represented by these peptides (e.g., A*24, DRB1*04:04, and DRB1*15:01) are covered by several other peptides, too. Two out of five peptides are found to be affected in their core region, which might influence the binding capability to the respective HLA alleles. A matching between patient and donor based only on these HLA alleles (B*27, DQB1*04) should therefore be avoided. Taken together, by adoptive transfer of human SARS-CoV-2-specific T cells isolated from convalescent individuals using MACS GMP PepTivator SARS-CoV-2 Select the protective T cell immunity in patients suffering from COVID-19 could be restored and may thereby prevent a severe course of the disease.

## Materials and Methods

### Convalescent COVID-19 Patients

The study was approved by the Internal Review Board of Hannover Medical School (MHH, approval number 3639_2017, 9001_BO-K, 9255_BO_K_2020). All 198 donors (n = 90 male, n = 108 female) aged 18–69 years (mean 41 years) were recruited from MHH between October 2020 and July 2021. Convalescent COVID-19 patients with confirmed history of SARS-CoV-2 infection were sampled up to 61 weeks after symptom onset and at least 2 weeks after negative PCR.

### Determination of Antiviral T Cells by ELISpot Assay

Virus-specific T lymphocytes were detected by Interferon-gamma (IFN-γ) Enzyme-Linked Immunospot (ELISpot) Assay as previously described (Bieling et al., 2018). Briefly, Peripheral Blood Mononuclear Cells (PBMC) were isolated from blood samples by discontinuous density gradient centrifugation, resuspended in culture medium consisting of RPMI-1640 (Lonza, Verviers, and Belgium) supplemented with 10% human AB serum (C.C.pro, Oberdorla, and Germany) at a concentration of 1 × 10^7^ cells/ml, seeded in 24-well plates and rested overnight. For retrospective analyses, PBMCs were frozen and stored at −70°C until thawing and overnight resting prior to ELISpot assay. Rested PBMCs were cultured in anti-IFN-γ pre-coated ELISpot plates (Lophius Biosciences, Regensburg, and Germany) for 16–18 h at a density of 2.5 × 10^5^ cells/well in presence of PepTivator SARS-CoV-2 membrane (M), nucleoprotein (N) or Select (Miltenyi Biotec, Bergisch Gladbach, Germany) or PepMix SARS-CoV-2 Spike (S) protein from wild type, Delta or Omicron VOC (JPT Peptide Technologies, Berlin, Germany) at a final concentration of 1 µg of each peptide/ml). PepTivator SARS-CoV-2 Select consists of 88 peptides with a length of 9–22 amino acids originating from structural and non-structural proteins. The peptides are restricted to Major Histocompatibility (MHC) class I (*n* = 63 peptides), covering *n* = 19 frequent Human Leukocyte Antigen (HLA) alleles, and MHC class II (n = 25), covering *n* = 6 frequent HLA alleles. Cells stimulated with staphylococcal enterotoxin B (1 μg/ml, SEB, Merck, Taufkirchen, Germany) served as positive controls, and PBMCs incubated in media alone as negative controls (NC). IFN-γ secretion was detected using streptavidin-alkaline phosphatase (Mabtech Stockholm, Sweden) and revealed by 5–13 bromo-4-chloro-3-indolyl phosphate/nitroblue tetrazolium (BCIP/NBT Liquid Substrate, Merck, Darmstadt, Germany). Spots were counted using AID ELISPOT 8.0 on an AID iSpot spectrum reader system (both from AID, Strassberg, and Germany). Means of duplicate well readings were calculated and expressed as the number of spots per well (spw). The threshold for a response was set at 2xNC+1 or ≥3.

### Characterization of Antiviral T Cells by Whole Blood Assay

The SARS-CoV-2 T Cell Analysis Kit (Whole Blood) (Miltenyi Biotec) was performed according to the manufacturer’s instructions. In brief, heparinized whole blood was filled into 48 well plates (1 ml/well) and stimulated with PepTivator SARS-CoV-2 Select (1 µg of each peptide/ml) for 8 h. Unstimulated samples served as negative control and samples stimulated with CytoStim (Miltenyi Biotec) served as positive control. Cytokine secretion was blocked by supplementation with Brefeldin A (Miltenyi Biotec). After a total of 8 h of stimulation, erythrocytes were lysed (Red Blood Cell Lysis Solution; Miltenyi Biotec), samples were fixed, and permeabilized followed by staining with the following antibodies: anti-CD3 Allophycocyanin (APC), anti-CD4 Vio Bright B515, anti-CD8 VioGreen, anti-IFN-γ Phycoerythrin (PE), anti-Tumor Necrosis Factor alpha (TNF-α) PE-Vio 770, anti-CD20 VioBlue, and anti-CD154 APC-Vio 770 (all Miltenyi Biotec). Samples were acquired on a MACSQuant Analyzer 10 (Miltenyi Biotec).

### Detection and Enrichment of Antiviral T cells by IFN-γ Cytokine Secretion Assay

The IFN-γ cytokine secretion assay (CSA, Miltenyi Biotec) was performed as previously described (Tischer et al., 2014a). After overnight resting in TexMACS Medium (Miltenyi Biotec), 1 × 10^7^ isolated PBMCs were stimulated with PepTivator SARS-CoV-2 Select (Miltenyi Biotec) at a final concentration of 1 µg of each peptide/ml peptide pool. Unstimulated PBMCs served as negative controls. Activated IFN-γ-secreting T cells were specifically captured during the magnetic enrichment process using anti-IFN-γ-PE antibodies and paramagnetic anti-PE microbeads. Aliquots of the respective cell fractions collected before (pre drug substance (preDS)) and after enrichment (DS) were used for analysis of IFN-γ^+^ T cell subsets by multicolor flow cytometry. Dead cells were excluded from analysis by 7-amino-actinomycin D (7-AAD) staining (BD Biosciences, Heidelberg, Germany). The percentage of viable IFN-γ^+^ cells was determined by staining the cells with anti-CD45 APC-H7, anti-CD3 Fluorescein Isothiocyanate (FITC), anti-CD8 APC, and anti-CD4 Alexa Fluor 700 (AF700) mAbs (all from BD Biosciences). At least 10,000 events were acquired in the viable CD45^+^ leukocyte gate in each test (system: FACSCanto10c, BD Biosciences, Heidelberg, Germany). CD3^+^/IFN-γ^+^, CD8^+^/IFN-γ^+^ and CD4^+^/IFN-γ^+^ T cell populations were gated based on the scatter properties of viable 7-AAD^-^/CD45^+^/CD3^+^ T cells. Moreover, for selected donors, the activation state of the cells was analyzed by staining the cells with anti-CD45 Pacific Blue, anti-CD3 FITC, anti-CD8 APC, anti-CD4 AF700, anti-CD14 BV510, anti-CD19 BV510, anti-CD69 BV605, anti-CD137 PE-Cyanine 7 (PE-Cy7), and anti-CD154 APC-Cy7 mAbs (BD Biosciences and BioLegend). Dead cells were excluded from analysis by 7-AAD staining (BD Biosciences). The following gating strategy was applied: CD45^+^7-AAD^-^SSC^low^CD3^+^CD14/19^-^ (CD3^+^ T cells), CD45^+^7-AAD^-^SSC^low^CD3^+^CD14/19^-^/CD8^+^ (CD8^+^ T cells), and CD45^+^7-AAD^-^SSC^low^CD3^+^CD14/19^-^/CD4^+^ (CD4^+^ T cells). The following acceptance criteria apply for donation of SARS-CoV-2-specific T cells for clinical application: CD3^+^/IFN-γ^+^ pre-enrichment ≥ 0.01% or enrichment of a clear, defined population of ≥10% CD3^+^/IFN-γ^+^.

### Gene Expression Analysis by Quantitative Real-Time PCR (qRT-PCR)

Messenger RNA (mRNA) amounts of IFN-γ, perforin and granzyme B were analyzed as previously described (Bunse et al., 2015). Briefly, total cellular RNA (RNeasy Mini Kit; QIAGEN, Hilden, Germany) was isolated from SARS-CoV-2-specific T cells magnetically enriched via CSA (see above) or unstimulated control cells. The cDNA was amplified using the High-Capacity cDNA Reverse Transcription Kit (Applied Biosystems, Darmstadt, and Germany). mRNA amounts were quantified using inventoried mixes and TaqMan Gene Expression Master Mix (both from Applied Biosystems). Constitutively expressed glyceraldehyde 3-phosphate dehydrogenase (GAPDH) served as the reference gene. All tests were performed in duplicates.

### Clinical Grade Manufacturing of SARS-CoV-2-specific T Cells

In *n* = 4 clinical-grade manufacturing processes, SARS-CoV-2-specific T cells were isolated from leukapheresis products (LP) of pretested COVID-19-recoved donors using the CliniMACS Prodigy device (Miltenyi Biotec) and the CliniMACS CCS IFN-gamma (Miltenyi Biotec) following the manufacturer’s written instructions. In brief, total nucleated cell numbers in the LP were measured (Coulter ACTdiff, Beckman Coulter, Krefeld, Germany) and the process was initiated using 1 × 10^9^ total nucleated cells. The following steps were carried out autonomously by the CliniMACS Prodigy device: *ex vivo* restimulation (4 h, 37°C, 5% CO_2_) with MACS GMP PepTivator SARS-CoV-2 Select (1 μg/ml per peptide, Miltenyi Biotec), labeling of white blood cells with the CliniMACS CCS Catchmatrix Reagent (37°C, 5% CO_2_), secretion phase, labeling and immunomagnetic separation of IFN-γ-secreting cells by antibody-conjugated super-paramagnetic particles (CliniMACS IFN-γ Enrichment Reagent, Miltenyi Biotec). Prior to immunomagnetic separation, a quality control sample (preDS) was taken from the quality control bag.

### Quality Control of Clinical-Grade SARS-CoV-2-specific T Cell Products

Quality control of LP, preDS, and DS was performed as previously described (Tischer et al., 2014b). Total CD45^+^ leukocytes, viability, and frequencies as well as total cell numbers were determined using Trucount Absolute Counting Tubes (BD Biosciences) and the following staining reagents: anti-CD45 APC-H7 and anti-CD3 FITC mAbs and 7-AAD (all BD Biosciences). After staining, erythrocytes were lysed using Lysing Solution (Beckman Coulter) and samples were acquired on a BD FACSCanto 10c with at least 10,000–50,000 events in the Trucount beads gate. Purity, memory phenotype and cellular composition were analyzed using combinations of the following staining reagents: anti-CD45 APC-H7, anti-CD3 FITC, anti-CD4 AF700, anti-CD8 APC, anti-CD14 BV510, anti-CD19 BV510, anti-IFN-γ PE, anti-CD45RA BV605, anti-CD62L BV421, anti-CD56 AF700, anti-CD33 APC, anti-CD19 PE-Cy7 mAbs, and 7-AAD (all BD Biosciences). After staining and lysis of erythrocytes, samples were acquired on a BD FACSCanto 10c with at least 10,000–50,000 events in the CD45^+^ leukocyte gate.

### Expansion of CCS-Enriched SARS-CoV-2-specific T Cells

SARS-CoV-2-specific T cells enriched via CCS using the CliniMACS Prodigy device and MACS GMP PepTivator SARS-CoV-2 Select (Miltenyi Biotec) were seeded into 24 well plates at 1 × 10^5^ cells per well in TexMACS (Miltenyi Biotec). For feeder cells, autologous PBMCs were irradiated at 50 Gy and seeded at 1 × 10^7^ cells per well. Cultures were supplemented with recombinant human Interleukin-2 (rhIL-2, 50 IU/ml; Miltenyi Biotec) and maintained for 7–13 days including regular media change and splitting of the cells.

### Cytotoxicity Assay

Autologous PBMCs were labeled using the CellTrace Violet Cell Proliferation Kit (Invitrogen, Waltham, MS United States), seeded into 24 well plates and loaded with PepTivator SARS-CoV-2 Select overnight. Unloaded PBMCs served as control for unspecific killing. Expanded T cells were harvested, counted and seeded into 96 well plates together with unloaded or loaded PBMCs in different effector-to-target ratios (1:1, 2:1, 5:1, and 10:1). As a control for target cell viability, target cells were incubated in absence of T cells. CellEvent Caspase 3/7 Detection Reagent (Invitrogen) was added to the cultures. After 4 hours, supernatants were collected for subsequent multiplex analysis. Cells were washed and stained with 7-AAD and analyzed via flow cytometry using the BD FACSCanto 10c system.

### Cytokine/Chemokine Measurement in Cell Culture Supernatant

Cell culture supernatants collected from cytotoxicity assays were subjected to LEGENDPlex using the CD8/NK Cell Kit (BioLegend). Samples were prepared and acquired using the BD FACSCanto 10c system according to the manufacturer’s instruction.

### Data Analysis

Data were analyzed using BD FACSDiva v8.0.1 (BD Biosciences), FlowJo v10 (FlowJo LLC, BD Biosciences), LEGENDPlex Software (v8.0), and Microsoft Excel 2010 (Microsoft Corporation, Redmond). Prism Version 8.2.0 (GraphPad Software, San Diego, United States) was used to generate graphs as well as for statistical analysis using a linear regression model and the Kruskal–Wallis or Friedman test followed by multiple comparison correction. Significance levels were calculated and expressed as *p*-values (*p < 0.05, **p < 0.01, ***p < 0.001, and ****p < 0.0001).

## Results

### Donor Screening and Pretesting for SARS-CoV-2-specific T Cells

**FIGURE 1:**
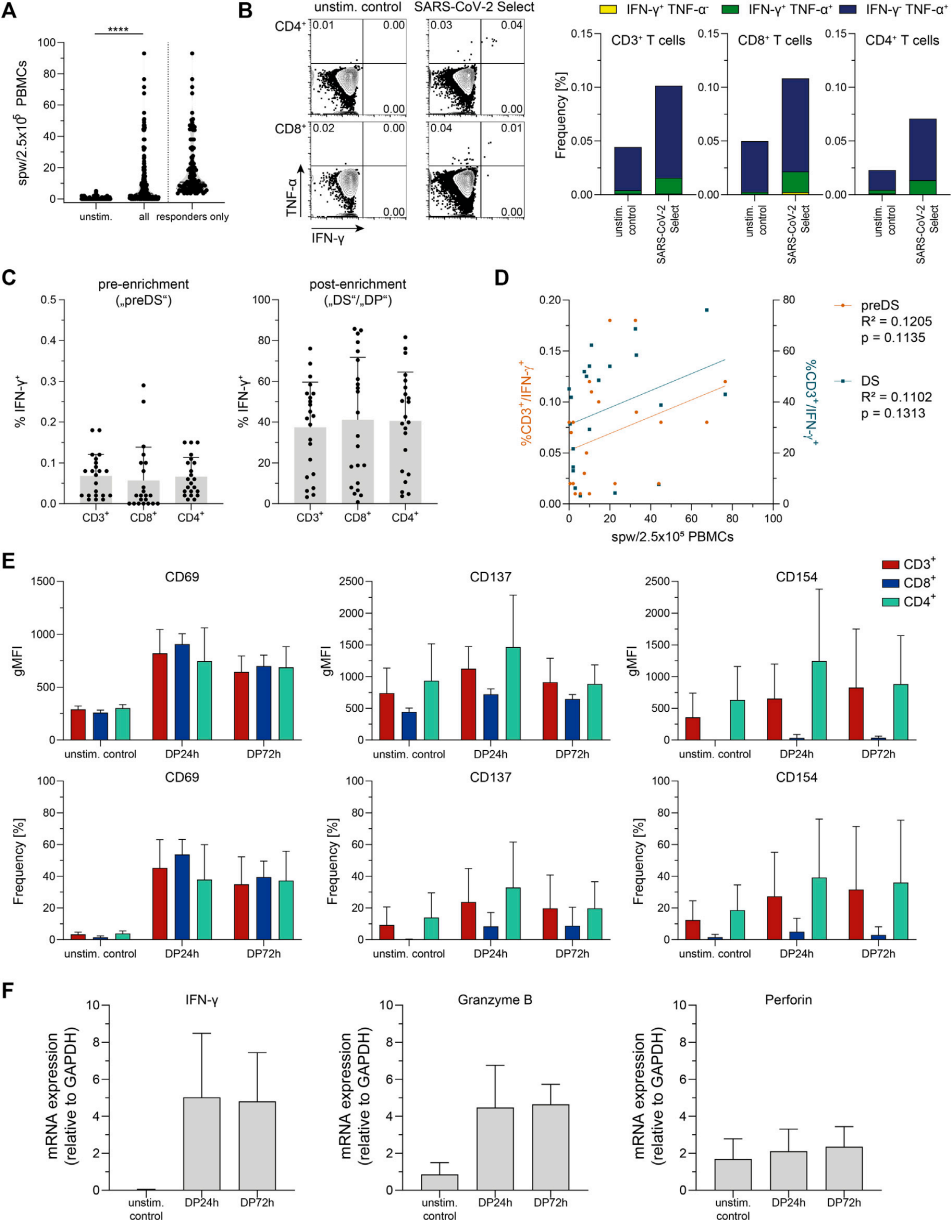
Donor screening and pretesting. **(A)** Summarized results of donor screening via Interferon-gamma (IFN-γ) Enzyme-Linked ImmunoSpot (ELISpot) assay, *n* = 198 (all) and *n* = 104 (responders only) convalescent COVID-19 patients. unstim.: unstimulated control; spw: spots per well. Data are shown as violin plots, each symbol represents data obtained from one donor. ****p < 0.0001; Wilcoxon matched-pairs signed rank test. **(B)** Representative FACS plots and summarized results from intracellular cytokine staining in whole blood after stimulation with PepTivator SARS-CoV-2 Select using the SARS-CoV-2 T Cell Analysis Kit (Whole Blood) (Miltenyi Biotec). unstim. control: unstimulated control; TNF-α: Tumor Necrosis Factor-alpha. Data are shown as mean of data obtained from *n* = 12 convalescent COVID-19 patients. **(C)** Summarized frequencies of IFN-γ-secreting T cell subsets detected and magnetically enriched using Cytokine Secretion Assay (CSA); left: pre-enrichment (corresponding to pre drug substance („preDS“) in clinical manufacturing), values obtained from unstimulated control were subtracted; right: post-enrichment (corresponding to „DS“/drug product („DP”) in clinical manufacturing). Data are shown as mean + SD of data obtained from *n* = 22 convalescent COVID-19 patients. Each symbol represents data obtained from one donor. **(D)** Correlation of data obtained from ELISpot Assay (spw/2.5 × 10^5^ PBMCs) and CSA (%CD3^+^/IFN-γ^+^) pre-enrichment (preDS) and post-enrichment (DS). Each dot corresponds to data obtained from one donor, *n* = 22 convalescent COVID-19 patients. Linear regression analysis was performed to calculate statistical significance. **(E)** Activation marker (CD69, CD137, and CD154) expression on indicated samples obtained via CSA, compared to unstimulated control (unstim. control). Data are shown as mean + SD of data obtained from *n* = 3 convalescent COVID-19 patients. **(F)** mRNA levels of IFN-γ, granzyme B, and perforin of indicated samples obtained via CSA. Data are shown as mean + SD of data obtained from *n* = 3 convalescent COVID-19 patients.

Convalescent COVID-19 donors (*n* = 198) were analyzed first with regard to antiviral T cell frequencies via IFN-γ ELISpot Assay using PepTivator SARS-CoV-2 Select (Figure 1A). SARS-CoV-2-specific T cells were detectable in n = 104/198 donors (52.5%) with a median frequency of 10.75 spw/2.5 × 10^5^ PBMCs (responders only). To further characterize the antiviral T cells, IFN-γ and TNF-α-producing T cells were detected in heparinized whole blood (Figure 1B). T cells responding to PepTivator SARS-CoV-2 Select were mainly multifunctional CD8^+^ and CD4^+^ cells producing IFN-γ and/or TNF-α (mean: CD8^+^ 0.06%; CD4^+^ 0.05% (cumulative), values from unstimulated control were subtracted).

In perspective of generation of SARS-CoV-2-specific T cells for clinical application, IFN-γ-secreting cells were detected and magnetically enriched via CSA (Figure 1C), representing the small-scale process largely analogous to the clinical large-scale CCS manufacturing process. In all tested donors (*n* = 22), SARS-CoV-2-specific T cells were detectable, with pre-enrichment frequencies of CD3^+^/IFN-γ^+^ cells ranging from 0.01 to 0.18% (mean 0.07%). Comparing the results obtained by ELISpot Assay and CSA, trends for a positive correlation were found between antiviral T cells detected by ELISpot Assay and frequencies of CD3^+^/IFN-γ^+^ cells both, pre-enrichment (corresponding to “preDS” in clinical-grade manufacturing) and post-enrichment (corresponding to “DS” in clinical manufacturing) (Figure 1D).

The mean purity obtained after magnetic enrichment was 37.44% (3.17–76.08%, Figure 1D). From the *n* = 22 donors tested, *n* = 18 fulfilled the acceptance criteria for donation of SARS-CoV2-specific T cells for clinical application (CD3^+^/IFN-γ^+^ pre-enrichment ≥ 0.01% or enrichment of a clear, defined population of ≥10% CD3^+^/IFN-γ^+^) with mean pre-enrichment frequencies of CD3^+^/IFN-γ^+^ cells of 0.08% (0.01–0.18%), and a mean purity obtained after magnetic enrichment of 44.58% (12.97–76.08%). In case of the remaining *n* = 4 donors, the enrichment of CD3^+^/IFN-γ^+^ T cells was below 10% (mean 5.33%; 3.17–7.62%). These donors did not fulfill the acceptance criteria, hence are not suitable for donation of SARS-CoV-2-specific T cells.

Small-scale T cell products obtained via CSA after magnetic enrichment were further characterized with respect to functionality as defined by activation state (Figure 1E) and expression of cytotoxic molecules (Figure 1F). Compared to the original fraction of unstimulated cells, the enriched T cell product displayed an activated phenotype as indicated by increased expression of activation-associated surface molecules CD69, CD137, and CD154. Upon storage for 72 h after sample collection, this activated phenotype did not further increase but slightly decrease yet remained at a level higher than observed in preDS, indicating intact T cell functionality up to 72 h after sample collection. Moreover, high expression levels of IFN-γ (mean 279-fold) and granzyme B mRNA (mean 5.2-fold) were detected in the enriched T cell products compared to unstimulated cells, while no difference was observed for perforin mRNA. The increased mRNA expression of IFN-γ and granzyme B was sustained until 72 h after sample collection.

In summary, antiviral T cells reactive against PepTivator SARS-CoV-2 Select were detectable in convalescent COVID-19 patients and these were mainly TNF-α and IFN-γ producing CD8^+^ and CD4^+^ T cells. The magnetic enrichment of SARS-CoV2-specific T cells using PepTivator SARS-CoV-2 Select and CSA resulted in T cell products with a mean purity of 44.58% (eligible donors only), an activated phenotype and expression of cytotoxic molecules. A positive correlation between results obtained by ELISpot Assay and CSA suggests the suitability of ELISpot Assay for screening of potential donors for antiviral T cell manufacturing (Tischer et al., 2014a). Together, these data give first evidence that the enrichment of SARS-CoV-2-specific T cells for clinical application using PepTivator SARS-CoV-2 Select and the cytokine capture approach is feasible.

### Clinical-Grade Manufacturing of SARS-CoV-2 Select-specific T Cells

**FIGURE 2:**
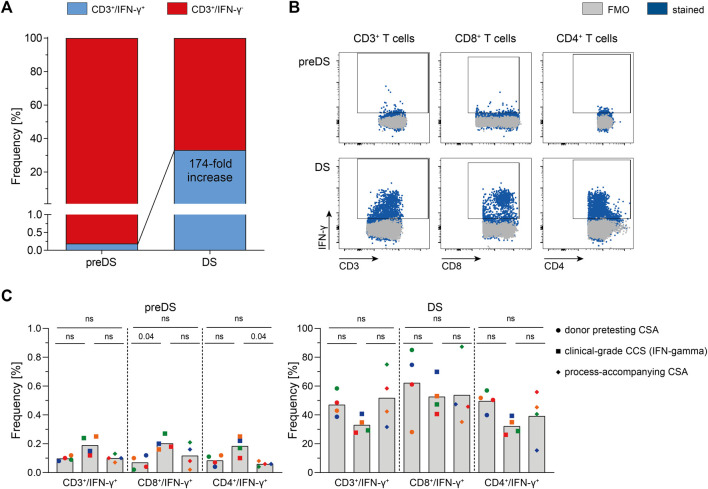
Clinical manufacturing of SARS-CoV-2-specific T cells (*n* = 4). SARS-CoV-2-specific T cells were magnetically enriched under GMP-compliant conditions using Cytokine Capture System (CCS) and CliniMACS Prodigy and analyzed via flow cytometry. **(A)** Enrichment of SARS-CoV-2-specific Interferon-gamma (IFN-γ)-secreting T cells using CCS and CliniMACS Prodigy. Data are shown as mean of data obtained from *n* = 4 manufacturing processes. **(B)** Representative FACS plots depicting IFN-γ production in indicated T cell subsets of in-process control (pre-enrichment; preDS) and the magnetically enriched T cell product (drug substance, DS). **(C)** Summarized results of clinical-grade manufacturing in comparison to corresponding donor pretesting Cytokine Secretion Assay (CSA) and process-accompanying CSA. Bars represent mean, each symbol represents data obtained from one donor (same colors indicate matched data), *n* = 4 convalescent COVID-19 patients. Statistical analysis was performed using Friedman Test, followed by Dunn’s multiple comparison; ns not significant.

Based on the results obtained via CSA using PepTivator SARS-CoV-2 Select, clinical-scale manufacturing processes using CliniMACS Prodigy, CCS IFN-gamma and MACS GMP PepTivator SARS-CoV-2 Select were performed. Donors (*n* = 4) were selected first based on results obtained via ELISpot Assay during donor screening and second based on results of CSA during donor pretesting. SARS-CoV-2-specific T cells responding to MACS GMP SARS-CoV-2 Select could be enriched by 174-fold (Figures 2A,B), resulting in a mean purity of 33.1% (Figure 2C). Clinical-grade manufacturing yielded cell numbers sufficient for the generation of a T cell product containing 5,000 CD3^+^/IFN-γ^+^ T cells per kg body weight for a patient with a body weight of up to 154 kg, demonstrating feasibility of SARS-CoV-2-specific T cell manufacturing. Comparison of donor pretesting CSA, clinical grade CCS IFN-gamma and process-accompanying CSA revealed largely similar frequencies of IFN-γ^+^ T cells both, pre-enrichment, and post-enrichment. In summary, these data show that CSA is currently the most suitable assay for donor selection because it can validly determine the antiviral T cell frequencies and–in contrast to other assays such as intracellular cytokine staining–predict the enrichment efficiency in the production process. Thus, this assay allows the selection of the best possible donor for the patient in that it is very likely that the manufacturing results in a good T cell product with respect to cell numbers and purity (Tischer et al., 2014b).

**FIGURE 3:**
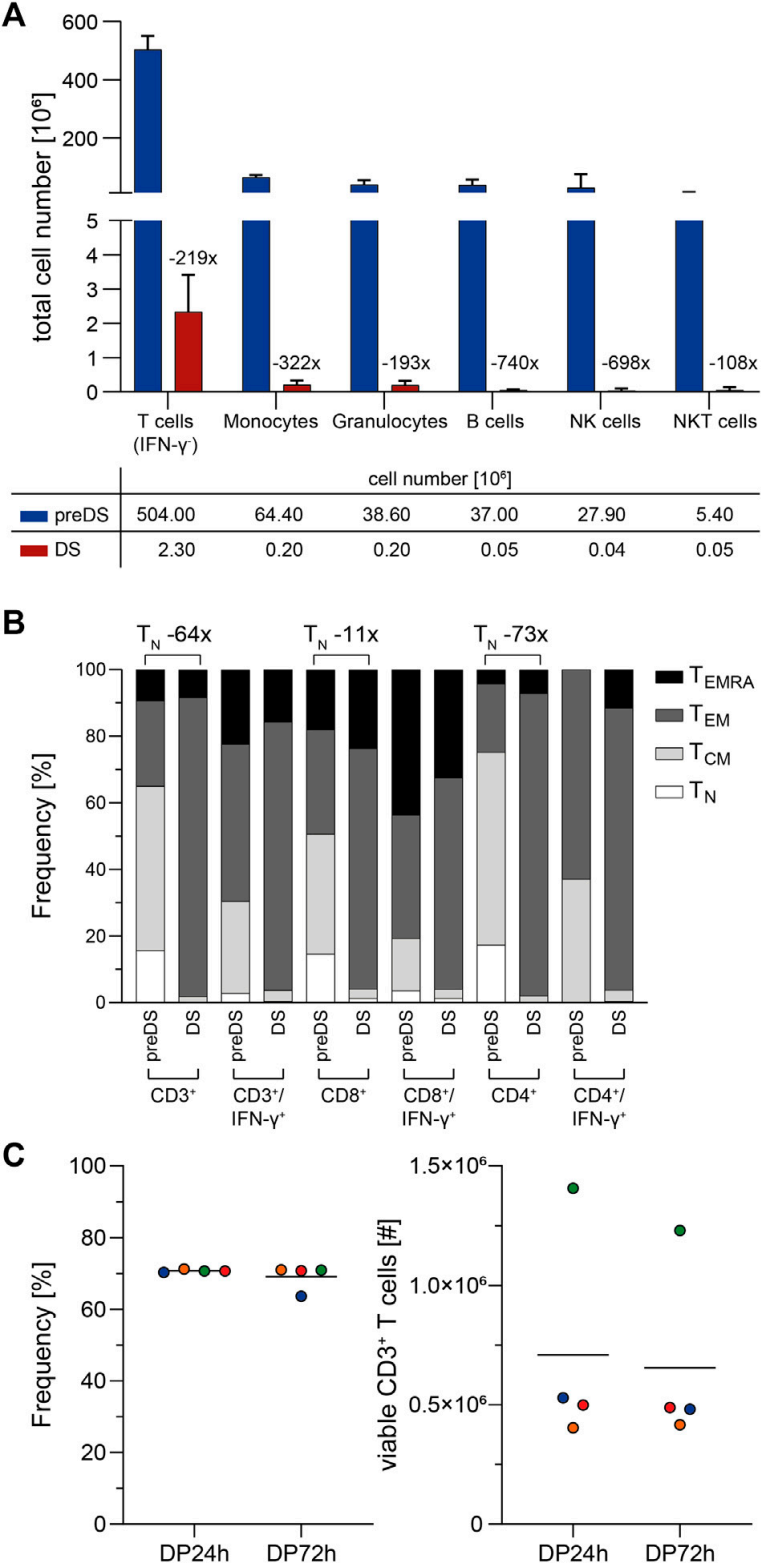
Characterization of clinical-grade SARS-CoV-2-specific T cells (*n* = 4). SARS-CoV-2-specific T cells were magnetically enriched under GMP-compliant conditions using Cytokine Capture System (CCS) and CliniMACS Prodigy and analyzed via flow cytometry. **(A)** Impurities of SARS-CoV-2-specific T cell product. Shown is the fold-reduction of indicated immune cell subsets in the T cell product (drug substance, DS) compared to preDS (pre-enrichment). Bars and lines represent mean and SD, *n* = 4 manufacturing runs. IFN-γ: Interferon-gamma; NK cells: Natural Killer cells, NKT cells: Natural Killer T cells. **(B)** Memory phenotype composition of T cell product (DS) compared to preDS. Numbers above bars indicate fold change of naïve T cells (T_N_) between DS and preDS within indicated T cell subsets. T_CM_: T central memory; T_EM_: T effector memory; T_EMRA_: T effector memory re-expressing CD45RA. **(C)** Stability of SARS-CoV-2-specific T cell product during shelf-life in terms of viability (left; Frequency of 7-AAD^-^ cells among CD45^+^ leukocytes) and total viable CD3^+^ T cell numbers (right) as determined via flow cytometry. 24 h/72 h: time after end of leukapheresis. Each symbol represents data obtained from one manufacturing run (same colors indicate matched data), horizontal lines represent the mean.

Analysis of cellular impurities in the DS revealed a decrease of CD3^+^/IFN-γ^-^ unspecific, potentially alloreactive T cells by 219-fold compared to preDS (Figure 3A). Moreover, innate immune cell subsets were decreased by 108- to 740-fold. Together with the decrease in unspecific CD3^+^/IFN-γ^-^ cells, frequencies of naïve T cells (T_N_) were reduced by 64-fold, while the frequencies of effector memory T cells (T_EM_) were markedly increased (Figure 3B). In perspective of clinical application, stability of the T cell product was analyzed (Figure 3C). Viability of the cells and total CD3^+^ T cell number was unaffected after 72 h of storage at 4°C after end of leukapheresis. The starting material (LP) and the final formulated T cell products (DP) were analyzed with respect to microbial sterility: No growth was observed within 7–14 days of incubation (data not shown). Endotoxin levels were assessed using Endosafe nexgen PTS and were <5 EU/ml (data not shown).

To estimate the reactivity of PepTivator SARS-CoV-2 Select-specific T cells towards the different VOCs, we performed ELISpot assay using peptide pools covering the Spike (S) protein from wild type (WT), and different VOCs as well as membrane (M) and nucleoprotein (N) (Supplementary Figure S1). Samples obtained at the time of leukapheresis (pre-vaccination) and recently collected samples (post-vaccination) were analyzed. All donors had initially been infected early in 2020 and have been vaccinated meanwhile. In accordance with previous reports, frequencies of T cells against M and N proteins slightly decreased throughout the time of convalescence, yet remained detectable (Gussarow et al., 2021). In contrast, T cell frequencies against PepTivator SARS-CoV-2 Select remained stable, suggesting an increased fraction of T cells against the S protein after vaccination. This is supported by the higher frequencies of WT S-specific T cells detected in the samples collected at a later time point. Of note and despite the fact that the donors were infected with the WT and all currently available vaccines are based on the sequences of WT S protein, T cell responses against Delta and Omicron VOCs were detectable both, in samples at the time of leukapheresis (pre-vaccination) as well as in the samples collected at a later time point (post-vaccination). Together, these data indicate that T cells enriched via cytokine capture approach using PepTivator SARS-CoV-2 Select contain T cells reactive against SARS-CoV-2 VOCs.

### Expansion Capacity and Cytotoxic Potential of Clinical-Grade Manufactured SARS-CoV-2-specific T Cells

**FIGURE 4:**
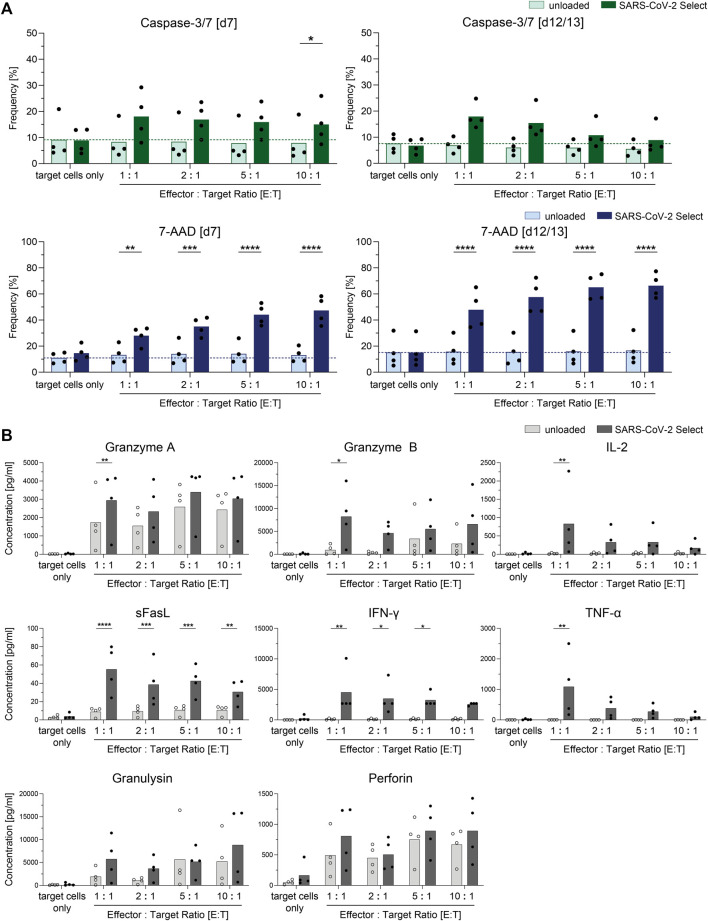
Cytotoxic potential of clinical-grade SARS-CoV-2-specific T cells (*n* = 4). SARS-CoV-2-specific T cells obtained by Cytokine Capture System (CCS) and CliniMACS Prodigy were expanded for 7–13 days and subjected to cytotoxicity assays using unloaded and SARS-CoV-2 Select-loaded autologous PBMCs as target cells. **(A)** Frequencies of cells with active caspase-3/7 or 7-AAD^+^ cells among target cells (CellTrace Proliferation dye positive). Light bars: unloaded target cells. Dark bars: loaded target cells. Results are displayed as individual results and as means. **(B)** Cell culture supernatants from cytotoxicity assays from day 12/13 were analyzed with respect to presence of cytotoxic effector molecules by LEGENDPlex Assay. Results are displayed as individual results and as means, *n* = 4 manufacturing processes. Statistical significance was calculated using Two-way ANOVA and Sidak’s multiple comparisons test. *p < 0.05, **p < 0.01, ***p < 0.001, and ****p < 0.0001.

SARS-CoV-2-specific T cells enriched by CCS IFN-gamma using the CliniMACS Prodigy System were expanded for up to 13 days using irradiated autologous PBMCs isolated from the LP and proved to maintain cellular function and high proliferative capacity (Figure 4). For determination of the isolated and expanded T cells’ capacity to recognize and kill target cells presenting peptides from the SARS-CoV-2 Select peptide pool, co-cultures of peptide pool-loaded or unloaded target cells and the expanded T cells were set-up. After 4 hours, the frequency of cells with active caspase-3/7 as well as the frequency of dead cells (7-AAD^+^) was determined using flow cytometry (Figure 4A). After 4 hours of co-cultivation, the antigen-loaded target cells displayed significantly increased frequencies of cells with active caspase-3/7 (Figure 4A). Moreover, the fractions of dead cells (7-AAD^+^) were significantly increased in co-cultures with antigen-loaded compared to unloaded target cells.

To investigate the cells’ capacity to produce and secrete cytotoxic mediators upon target recognition, cell culture supernatants of co-cultures with target cells loaded with PepTivator SARS-CoV-2 Select were analyzed via LEGENDPlex assay for secretion of effector molecules (Figure 4B). Significantly increased concentrations of granzyme A, granzyme B, IL-2, sFasL, IFN-γ, and TNF-α were detected in the cell culture supernatant of co-cultures with SARS-CoV-2 Select-loaded target cells when compared to unloaded target cells, indicating cytotoxic potential of the cells.

## Discussion

SARS-CoV-2-specific T cells developing during infection and subsequent recovery from the disease support viral clearance and control of infection. The developing antiviral T cell repertoire covers a broad range of viral antigens including both, structural and non-structural proteins. COVID-19 patients with insufficient endogenous functional antiviral T cells might benefit from adoptive transfer of SARS-CoV-2-specific T cells isolated from convalescent individuals, a concept which has been developed for viral complications with CMV, ADV, and EBV in immunosuppressed patients after transplantation (Kaeuferle et al., 2019). This study aimed to establish a screening and pretesting strategy to select for potential suitable T cell donors as well as proving feasibility of a rapid clinical-grade SARS-CoV-2-specific T cell enrichment using the CliniMACS Prodigy, CCS IFN-gamma, and MACS GMP PepTivator SARS-CoV-2 Select. In this line, the existing allogeneic T cell registry alloCELL (http://www.alloCELL.org) of the Institute of Transfusion Medicine and Transplant Engineering at Hannover Medical School has been expanded to SARS-CoV-2-specific T cells in donors recovered from SARS-CoV-2 infection. Memory T cell responses against SARS-CoV-2 were quantified and characterized after antigenic restimulation using ELISpot assay, intracellular cytokine staining, and quantitative realtime PCR. Furthermore, in light of clinical-grade manufacturing for adoptive T cell therapy, potential T cell donors were pretested using Cytokine Secretion Assay, largely analogous to the clinical-scale enrichment process via CCS IFN-gamma, and CliniMACS Prodigy. In contrast to ELISpot assay and intracellular cytokine staining, CSA allows for enrichment of antigen-specific T cells secreting IFN-γ upon stimulation. Moreover, frequencies of IFN-γ^+^ T cells obtained via CSA were comparable to those obtained during clinical-grade manufacturing, demonstrating that CSA is well-suited for selection of potential T cell donors. The four generated clinical-grade T cell products were characterized with respect to their cellular composition as well as to their proliferative and cytotoxic potential by using flow cytometry, *in vitro* cytotoxicity assays and multiplex analysis. In summary, these data demonstrate that the enriched clinical-grade SARS-CoV-2-specific T cells are able to replicate, specifically recognize and kill target cells, and secrete cytotoxic molecules upon target recognition.

One of the most dreaded side effects of the administration of T cell products is the development of Graft-versus-Host Disease (GvHD) triggered by alloreactive T cells in the product. The presence of naive T cells in T cell products precipitates the risk of GvHD, since naive T cells have a typically broader T cell receptor (TCR) repertoire and, therefore, a higher alloreactivity potential than memory T cell fractions (Nikolich-Zugich et al., 2004; Inman et al., 2018). In a single center dose escalation study in Spain, CD45RA-depleted, unselected memory T cells from COVID-19 convalescent donors were given at increasing doses of 1 × 10^5^, 5 × 10^5^, and 1 × 10^6^ memory T cells/kg body weight to hospitalized COVID-19 patients at risk for a severe course (Perez-Martinez et al., 2021; Pérez-Martínez et al., 2021). All three dose levels were tolerated without adverse events. Clinical status of the patients, as assessed by the National Early Warning Score and the 7-point WHO ordinal COVID-19 scale, improved 6 days after application of the Investigational Medicinal Product (IMP). The results from this study suggest that hospitalized COVID-19 patients at risk for a severe course might benefit from adoptive transfer of antiviral memory T cells.

In contrast to CD45RA-depleted memory T cell products, the cellular products generated in this study are specifically enriched for SARS-CoV-2 memory T cells. The recognition of infected cells by enriched antiviral T cells via the corresponding epitopes will lead to the generation and proliferation of endogenous virus-specific T cells in the patient and thus successfully and permanently control the virus. It has recently been shown that third party donor-derived T cells had contributed to endogenous CMV-specific T cell reconstitution in a patient (Neuenhahn et al., 2017; Fabrizio et al., 2021). Therefore, also transferred matched CD4^+^ T cells harbor the potential to successfully contribute to the elimination of the virus and the infected cells by inducing endogenous CD8^+^ T cells or vice versa. The effectiveness of CD4^+^ T cell responses has been demonstrated for AdV (Sester et al., 2002) and polyomavirus BKV (Espada et al., 2020) as well as for SARS-CoV-2 (Grifoni et al., 2020) and is dominated by CD4^+^ T cells with memory/effector phenotype. On the other hand, the transfer of single-matched HLA-class I-restricted antiviral T cells was sufficient to control CMV infection or reactivation in patients (Uhlin et al., 2012; Grifoni et al., 2020). Antiviral CD4^+^ and CD8^+^ T cells recognize virus-infected cells via the presentation of viral peptides in context of HLA class I or II molecules and are activated by interaction of their TCR with the specific peptide/HLA complexes on the virus-infected cells. The antiviral T cells proliferate and CD4^+^ T cells secrete cytokines in order to support the proliferation and function of CD8^+^ T cells, thereby contributing to prolonged survival of the adoptively transferred CD8^+^ T cells (Kaeuferle et al., 2019). The interaction between CD4^+^ and CD8^+^ T cell populations is crucial for the elimination of virus-infected cells. Therefore, donor selection for SARS-CoV-2-specific T cell therapy should strongly consider the (partial) match in HLA class I and II alleles of recipient and donor.

Leung and colleagues reported successful manufacturing of SARS-CoV-2-specific T cells from six convalescent COVID-19 patients using CCS IFN-gamma and peptide pools PepTivator SARS-CoV-2 M, N, and S (Leung et al., 2020). They hypothesize that the probability that the recipient will share at least one HLA allele with one of the six donors will be between 88 and 99%, depending on ethnic background. The peptide pool used in this study, PepTivator SARS-CoV-2 Select, consists of 88 peptides with a length of 9–22 amino acids originating from structural and non-structural proteins. Of these, 63 peptides are restricted to HLA class I and 25 peptides are restricted to HLA class II. Overall, the selected peptides allow a broad HLA coverage throughout the Caucasian population and stimulate both, CD4^+^ and CD8^+^ T cell subsets. In fact, the HLA-A, -B, and DR alleles represented in the SARS-CoV-2 Select peptide pool are present in >95%, >85% and >90% of the donors in the alloCELL registry, respectively. Of note, the majority of these peptides are identical in wild type and currently circulating VOCs, including the Delta and Omicron variants. It is assumed that future emerging VOCs will be sufficiently covered as well by PepTivator SARS-CoV-2 Select. Here, we were able to show that a large fraction of convalescent COVID-19 patients has specific T cells reactive against PepTivator SARS-CoV-2 Select and that these T cells could be magnetically enriched to high purities via IFN-γ CSA.

In large-scale GMP-compliant manufacturing runs using CliniMACS Prodigy, clinical-grade T cells products with purities comparable to those obtained in small-scale donor pretesting were achieved. To minimize the risk of GvHD, the overall T cell dose for adoptive transfer including virus-specific and contaminating T cells should be limited to total 25,000 CD3^+^ T cells/kg body weight, corresponding to the upper limit of T cells of donor lymphocyte infusion (DLI) in the haploidentical setting deemed to be safe (Lewalle et al., 2003; Rettinger et al., 2011; Quaiser and Köhl, 2018). Virus-specific T cells manufactured using CliniMACS Prodigy and CCS IFN-gamma enriches for memory T cells and reduces the number of naïve T cells, which could be confirmed by the results of this study. Assuming that naive T cells are mainly responsible for GvHD, enrichment of antiviral T cells by IFN-γ secretion will significantly lower GvHD risk while preserving immune memory against viral infections (Teschner et al., 2014; Bleakley et al., 2014; Bleakley et al., 2022). This is further supported by two literature reports (Feuchtinger et al., 2006; Aïssi-Rothé et al., 2010) showing that enrichment for virus-specific T cells reduced proliferation stimulated by a mixed lymphocyte culture with HLA mismatched PBMCs by one to two logs compared with the original lymphocyte harvest. While COVID-19 patients may be less prone to GvHD as they are not fully immunodeficient, on the other hand they may be more prone to GvHD as they have a hyperinflammatory milieu (García, 2020). Hence, for safety reasons a dose finding study seems advisable. In this regard, a total CD3^+^ T cell dose of no more than 25,000 cells per kg body weight is 4- to 40-times lower than the CD45RA-depleted memory T cells from COVID-19 convalescent donors applied in the single center dose escalation study, where no toxicities or adverse events were observed (Pérez-Martínez et al., 2021). In contrast to CD45RA-depleted memory T cell products, the cellular products generated in this study are enriched for SARS-CoV-2 memory T cells, therefore resulting in a product specific for COVID-19 patients. In the four manufacturing processes performed in this study, on average cell numbers sufficient for treatment of a patient of 154 kg body weight receiving a dose of 5,000 CD3^+^/IFN-γ^+^ cells per kg body weight and without exceeding the limit of 25,000 total CD3^+^ T cells per kg body weight were obtained. Moreover, the antiviral T cells were able to proliferate and specifically recognize target cells presenting SARS-CoV-2-specific peptides. Recognition of target cells resulted in secretion of cytotoxic molecules as well as induction of caspase activity and cells death of target cells. Therefore, the adoptively transferred enriched antiviral T cells are expected to be more efficient in clearance of the SARS-CoV-2 infection compared to CD45RA-depleted memory T cells. The target population for the first phase I clinical trial in Germany (ClinicalTrials.gov Identifier: NCT04762186) are patients with COVID-19, WHO clinical progression scale 4 with predefined risk factors for a severe disease course or patients with WHO clinical progression scale 5 (WHO Working Group on the Clinical Characterisation and Management of COVID-19 infection, 2020).

## Conclusion

The cellular immune response against SARS-CoV-2 is characterized by antiviral T cells with a broad repertoire, remaining readily detectable more than 1 year after initial infection. In contrast to antibody generation the protective effect of this T cell response is largely maintained also towards viral VOCs. Immunodeficient individuals or COVID-19 patients with insufficient endogenous T cell responses might therefore benefit from adoptive transfer of allogeneic, SARS-CoV-2-specific T cells isolated from convalescent individuals. In the present study, we have shown that PepTivator SARS-CoV-2 Select covering a broad variety of structural and non-structural SARS-CoV-2 proteins is suitable for donor selection and subsequent clinical-scale manufacturing of SARS-CoV-2-specific T cells. We performed four successful clinical-grade manufacturing processes for the generation of SARS-CoV-2-specific T cell products for adoptive T cell therapy. Enriched SARS-CoV-2-specific T cells harbored proliferative capacity and cytotoxic potential towards their target cells. By enriching for antiviral memory T cells with depletion of potentially alloreactive naïve T cells and careful consideration of partial HLA class I and II matching between donor and recipient, adoptive transfer of SARS-CoV-2-specific T cells should provide a unique and safe treatment option for COVID-19 patients at risk for a severe course of the disease.

## Data Availability

The original contributions presented in the study are included in the article/Supplementary Material, further inquiries can be directed to the corresponding author.

## Footnotes

1
https://www.fda.gov/news-events/press-announcements/coronavirus-covid-19-update-fda-authorizes-additional-oral-antiviral-treatment-covid-19-certain.

2
https://www.fda.gov/news-events/press-announcements/coronavirus-covid-19-update-fda-authorizes-first-oral-antiviral-treatment-covid-19.

3
https://www.ema.europa.eu/en/human-regulatory/overview/public-health-threats/coronavirus-disease-covid-19/treatments-vaccines/treatments-covid-19/covid-19-treatments-under-evaluation#covid-19-treatments-under-marketing-authorisation-evaluation-section.

4
https://outbreak.info/situation-reports.
