# Supplementary material for: Rapid Manufacturing of Highly Cytotoxic Clinical-Grade SARS-CoV-2-specific T Cell Products Covering SARS-CoV-2 and Its Variants for Adoptive T Cell Therapy
Source: Front Bioeng Biotechnol. 2022 Apr 4;10:867042. doi: 10.3389/fbioe.2022.867042 (PMC9036989; doi:10.3389/fbioe.2022.867042)
Supplement: Supplementary file 1 [file Image1.pdf]

Supplementary Material

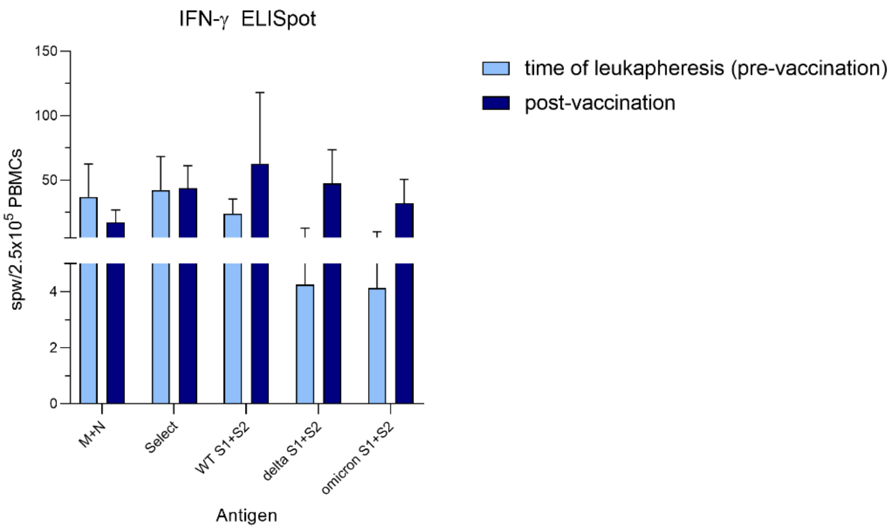

Supplementary Figure 1. Antiviral T cells against SARS-CoV-2 wild type (WT) and variants of concern (VOC) in convalescent COVID-19 patients. PBMCs obtained at the time of donor pre-testing (pre-vaccination) and PBMCs isolated from recently collected samples (post-vaccination) were analyzed via Interferon-gamma (IFN-γ) Enzyme-Linked ImmunoSpot (ELISpot) assay using peptide pools covering the Spike (S) protein from WT and different VOCs as well as membrane (M) and nucleoprotein (N). Data are shown as mean+SD. spw: spots per well.
